# MiR-1278 targets CALD1 and suppresses the progression of gastric cancer via the MAPK pathway

**DOI:** 10.1515/med-2023-0776

**Published:** 2023-11-18

**Authors:** Jia-Bei Xie, Hao Zhang, Xiao-Fang Li, Shuang-Yin Han, Xiu-Ling Li

**Affiliations:** Department of Gastroenterology and Hepatology, Henan Provincial People’s Hospital, People’s Hospital of Zhengzhou University, No. 7 Weiwu Road, Jinshui District, Zhengzhou 450003, Henan, China; Department of Gastroenterology and Hepatology, Henan Provincial People’s Hospital, People’s Hospital of Zhengzhou University, Zhengzhou 450003, Henan, China

**Keywords:** miR-1278, CALD1, gastric cancer, migration

## Abstract

This study aimed to investigate the interaction between miR-1278 and Caldesmon (CALD1) in gastric cancer (GC) and the regulatory mechanism. In both GC cells and tissues, the levels of CALD1, miR-1278, migration-related markers (E-cadherin, N-cadherin, and Snail), and MAPK signaling pathway-related proteins were clarified using quantitative real-time PCR and western blotting analyses. The effects of miR-1278 and CALD1 on GC cell viability and migration were analyzed using CCK-8 and Transwell assays, respectively. The targeting effect of miR-1278 on CALD1 was investigated using bioinformatics prediction and a dual luciferase reporter assay. The effect of miR-1278 on tumor growth was estimated *in vivo* using a tumor xenograft assay. In GC, miR-1278 expression decreased, whereas CALD1 was highly expressed. Transfecting an miR-1278 mimic into cells inhibited the viability as well as migration of GC cells, and suppressed Ras, phosphorylated (p)-P38, and p-ERK1/2 protein levels. Moreover, miR-1278 targeted and negatively regulated CALD1 expression. CALD1 overexpression promoted GC cell survival and migration and activated the MAPK pathway. Treatment with an miR-1278 mimic partially rescued the changes caused by CALD1 overexpression. Overall, our study revealed that miR-1278 suppresses the malignant behavior of GC cells by targeting CALD1 and regulating the MAPK pathway.

## 1 Introduction

Gastric cancer (GC) is the sixth most common cancer in both men and women with a worldwide prevalence rate of 11.1/100,000 population in 2020 [1]. Although great achievements in the surgical treatment and prognosis of GC have been noted, the overall 5-year survival rate of patients with GC remains low, at approximately 31% [2]. Studies have shown that the occurrence of GC is associated with risk factors such as smoking, alcohol abuse, irregular diet, *Helicobacter pylori* infection, and genetic factors [3]. However, the pathogenesis of GC is still unclear. Therefore, exploring new diagnostic biomarkers for GC is of great significance for the diagnosis and treatment of GC.

microRNAs (miRNAs) are endogenous small non-coding RNAs that can regulate key processes, including proliferation, invasion, differentiation, apoptosis, as well as metastasis, through post-transcriptional regulation, and play a key regulatory role in tumor development. Various abnormally expressed miRNAs have been identified in GC, such as miR-21, which is overexpressed in both human GC tissues and cell lines and acts as an oncogenic miRNA, thereby enhancing GC cell proliferation and invasion [4,5,6]. Meanwhile, miR-451, miR-486, and miR-101 [7,8,9] can act as either oncogenic miRNAs or tumor suppressors. Our study observed the upregulation of miR-1278 in GC. Previous studies have suggested that miR-1278 is considerably enriched in thyroid cancer, promotes cancer cell growth, migration, and invasion, and may function as an oncogenic miRNA [10]. In colorectal cancer, miR-1278 can also function as a potential biomarker and act as a tumor suppressor [11]. However, the role of miR-1278 in GC remains unclear; in particular, its role requires further investigation due to its abnormal expression in GC.

Caldesmon (CALD1) is a cytoskeleton-associated protein that has multiple isoforms and plays multiple regulatory roles. It has been shown that CALD1 plays a key regulatory role in the growth, migration, invasion, and apoptosis of tumor cells [12], and enrichment of CALD1 is linked to poor metastasis and prognosis in different cancers, including gastric, bladder, and colorectal cancers [13,14]. In particular, CALD1 was found to be strongly related to several immune as well as stromal components of the tumor microenvironment [15], and CALD1 levels in GC were closely associated with infiltration of immune cells, including dendritic cells, T cells, and tumor-associated macrophages [16]. Meanwhile, various functions regulated by CALD1, such as cell adhesion and leukocyte transendothelial migration, are closely related to the MAPK and TGF-β signaling pathways, which play a crucial role in both the proliferation and migration of tumor cells [17]. Therefore, CALD1 acts as a key regulator of GC progression; however, the specific regulatory mechanism requires further investigation.

In the current study, we focused on the roles and mechanisms of action of miR-1278 and CALD1 in GC. We hypothesized that miR-1278 could target and negatively regulate CALD1 expression and affect GC cell survival and migration by regulating the MAPK signaling pathway. Our results suggest that miR-1278 is a potent diagnostic marker for the treatment of GC patients, and the miR-1278/CALD1 axis is a therapeutic target for GC.

## 2 Methods

### 2.1 Patient sample collection

All GC tissue specimens and the corresponding paracancerous tissues (normal gastric mucosal tissues, 5 cm from the tumor margin) were obtained from patients with GC. The patients did not receive other therapies, such as radiotherapy or chemotherapy, before surgery. We obtained 38 matched normal and tumor samples. Tissues were confirmed as GC by at least three pathologists.

### 2.2 Cell culture

Human GC cell lines (HGC-27, AGS, and GTL-16) and the immortalized gastric epithelial cells (GES-1) were purchased from the Chinese Academy of Sciences Cell Resource Center (Shanghai, China). All cell lines were routinely cultured in RPMI 1,640 medium (Gibco, USA) at 37°C in an incubator with 5% CO_2_. Cell passaging was performed at a split ratio of 1:3 to 1:4, and the frequency of fluid exchange was three times per week.

### 2.3 RNA extraction and quantitative real-time PCR (qRT-PCR) assay

RNA isolation was performed using RNAiso Plus (TAKARA, Japan), and a Reverse Transcription kit (Invitrogen, USA) was used for cDNA generation. The miRNEasy EFPE Kit (BioTeke, China) was utilized to extract miRNA, followed by the generation of cDNA using the TaqMan miRNA reverse transcription kit (Applied Biosystems, USA). Next, PCRs were performed using the TaqMan Fast Universal PCR Master Mix (Applied Biosystems) and Hieff qPCR SYBR Green Master Mix kit (Yeasen, China) on ABI 7500 PCR (Applied Biosystems). The ΔCt values were calculated for each group, and gene levels were determined using the 2^–ΔΔCt^ method; the values were normalized using U6 (internal reference of miR-1278) or GAPDH (internal reference of CALD1) as internal controls. The primers used in this study are listed in Table 1.

**Table 1:** Primer sequences for qRT-PCR

Gene	Primer sequence (5′–3′)
miR-1278	Forward primer: GGCTCTGGCTCCGTGTCTT
	Reverse primer: CAGTGCAGGGTCCGAGGTATT
CALD1	Forward primer: TGGAGGTGAATGCCCAGAAC
	Reverse primer: GAAGGCGTTTTTGGCGTCTTT
U6	Forward primer: CAAATTCGTGAAGCGTTCCATA
	Reverse primer: AGTGCAGGGTCCGAGGTATTC
GAPDH	Forward primer: GGAGCGAGATCCCTCCAAAAT
	Reverse primer: GGCTGTTGTCATACTTCTCATGG

### 2.4 Cell transfection

The CALD1 overexpression vector (CALD1-OE) and empty vector were synthesized by Agilent Technologies (USA). We obtained an miR-1278 mimic and a mimic negative control (mimic-NC) from SwitchGear Genomics (USA). Next, Lipofectamine 3000 (Invitrogen) reagent was used to transfect mimic (75 nM) or vector (2 μg/mL) into AGS and GTL-16 cells, following the manufacturer’s protocol. Two days later, both cell types were seeded for qRT-PCR analysis.

### 2.5 Western blotting

From each group, total protein was extracted from cells using a lysis buffer (Beyotime, China), followed by quantification using a BCA kit (Pierce, USA). Next, 30 μg protein was isolated using SDS-PAGE (12%), and proteins were transferred to poly(vinylidene fluoride) (PVDF) membranes (Millipore, USA). Blocking was performed by dipping the membranes in 5% skimmed milk for 2 h at room temperature, followed by overnight incubation of protein bands at 4°C in the corresponding primary antibody: E-cadherin (cat. no. abs130068; Absin, China; 1:1,000), N-cadherin (cat. no. abs155766; Absin; 1:1,000), Snail (cat. no. abs151371; Absin; 1:1,000), Ras (cat.no. 91054; Cell Signaling Technology, USA; 1:1,000), P38 (cat.no. 8690; Cell Signaling Technology; 1:1,000), phosphorylated (p)-P38 (cat.no. 4511; Cell Signaling Technology; 1:1,000), ERK (cat.no. 4695; Cell Signaling Technology; 1:1,000), p-ERK (cat.no. 4370; Cell Signaling Technology; 1:2,000), and GAPDH (cat.no. 51332; Cell Signaling Technology; 1:1,000). Subsequently, the PVDF membranes were incubated with the corresponding secondary HRP-linked mouse or rabbit antibody (Cell Signaling Technology; 1:1,000) for 1 h at room temperature. Band images were visualized using enhanced chemiluminescence (Thermol Biotech Inc, USA).

### 2.6 CCK-8 assay

Cells were seeded in 96-well plates at 5,000 cells/well, and 3 replicate wells were set for each group. The cells were then cultured at 0, 24, 48, and 72 h and mixed with 10 μL of CCK-8 solution (Biosharp, China) at 37°C for 1 h. The absorbance at 450 nm was measured using an ElX-800 absorbance spectrophotometer (Bio-Tek, USA).

### 2.7 Transwell assay

Cell migration and invasion were detected after different transfections using a Transwell assay. In brief, 1  ×  10^5^ cells were added in the upper compartment of Transwell chambers (pore size, 8 μm) (Corning, USA). Next, 600 μL of complete medium containing 10% FBS was added to the lower compartment. After incubation for 24 h, cells in the upper compartments were removed with cotton buds, and the cells translocated to the lower compartments were fixed in 95% ethanol for 5 min and stained with 4 g/L crystalline violet solution (Biyuntian Biotechnology, China) for 1 h. Migratory cells were counted under the microscope (magnification 100×).

To estimate cell invasion, the upper compartment of Transwell chambers was first covered with Matrigel overnight at 4°C before seeding cells. The remaining experimental steps were the same as those of the migration experiment.

### 2.8 Tumor xenografts in nude mice

Ten male BALB/c nude mice, aged 4–5 weeks, were purchased from Beijing Spefford (China) and housed in individually ventilated cage boxes in a class 10,000 barrier environment by qualified and accredited animal managers. Mice were randomly assigned into the agomir-NC and agomir groups (five mice per group). Agomir-NC as well as agomir-transfected AGS cells were injected into mice via the subcutaneous route (2 × 10^6^ cells resuspended in 200 μL of PBS). Tumor formation was observed every 4 days by measuring the length and width of the formed tumors. The mice were euthanized on day 28; the tumors were removed intact, and the wet weight of each tumor was examined.

### 2.9 Dual-luciferase reporter assay

In 24-well plates, the AGS cells and GTL-16 cells were inoculated at a density of 5 × 10^4^ cells/well. According to the binding sites between miR-1278 and CALD1 predicted by starBase, we inserted the CALD1 3′ untranslated region (UTR)-wild-type (wt) and mutated type (mut1, mut2, or co-mut) downstream of pGL3.0 vectors (Promega, USA) to manufacture the luciferase expression vectors. miR-1278 mimic or miR-NC was co-transfected along with luciferase expression vectors into AGS and GTL-16 cells using Lipofectamine 2000. After 48 h of transfection, we measured the relative luciferase activity using a dual luciferase reporter gene assay (Promega, USA).

### 2.10 Statistical analysis

SPSS 22.0 statistical software was used to analyze the data. Three parallel experiments were set up for each group, with three independent replications. The data are expressed as the mean ± standard deviation. We used Student’s *t*-test for independent samples for comparison between different groups and one-way analysis of variance for multiple-group comparisons. In GC tissues, Pearson’s analysis was performed to evaluate the correlation between miR-1278 and CALD1 expression. *p* < 0.05 was considered to indicate statistically significant differences.


**Ethics approval and consent to participate:** The present study was approved by the Ethics Committee of Henan Provincial People’s Hospital (Zhengzhou, China). The processing of clinical tissue samples is in strict compliance with the ethical standards of the Declaration of Helsinki. All patients signed a written informed consent. All animal experiments comply with the ARRIVE guidelines and were carried out in accordance with the National Research Council’s Guide for the Care and Use of Laboratory Animals. The procedures performed in this animal study had an approval from the Animal Ethics Committee of Henan Provincial People’s Hospital (Zhengzhou, China).
**Consent for publication:** Consent for publication was obtained from the participants.

## 3 Results

### 3.1 miR-1278 expression is downregulated in GC, and overexpression of miR-1278 inhibits viability and migration of GC cells and suppressed the MAPK signaling pathway *in vitro*

**Figure 1:**
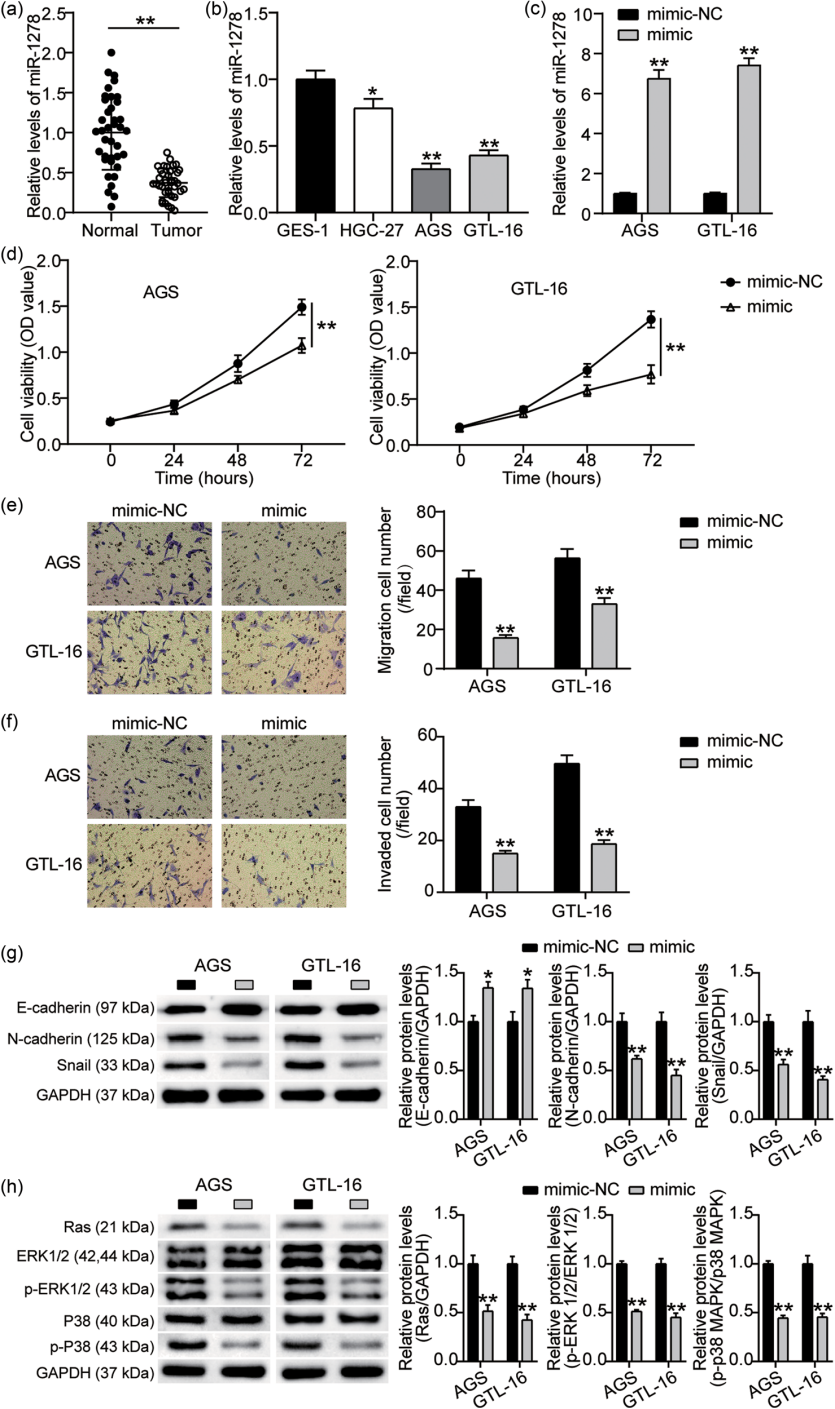
miR-1278 is downregulated in GC, whereas miR-1278 overexpression inhibits the viability and migration of GC cells and suppresses the MAPK signaling pathway *in vitro.* (a) qRT-PCR analysis of miR-1278 levels in GC tissues and paracancerous tissues (**P < 0.001). (b) The levels of miR-1278 in GC cells (HGC-27, AGS, GTL-16) and gastric epithelium immortalized cells (GES-1) were determined using qRT-PCR (compared with the GES-1 group, *P < 0.05 and **P < 0.001). (c) Transfection efficiency of the miR-1278 mimic and mimic-NC in GC cells, measured using qRT-PCR (compared with the mimic-NC group, **P < 0.001). (d) The effects of miR-1278 overexpression on GC cell viability were analyzed using a CCK-8 kit (compared with the mimic-NC group, **P < 0.001). (e and f) The effects of miR-1278 overexpression on the migration and invasion of GC cells were analyzed using a Transwell assay (compared with the mimic-NC group, **P < 0.001). (g) The regulation of migration-related protein markers (E-cadherin, N-cadherin, and Snail) by miR-1278 overexpression was analyzed using western blotting. (h) The regulation of MAPK signaling pathway-related protein levels (Ras, P38, p-P38, ERK1/2, and p-ERK1/2) by miR-1278 overexpression was analyzed using western blotting (compared with the mimic-NC group, **P < 0.001).

To investigate the role of miR-1278 in GC, we first collected samples and determined miR-1278 expression levels in tumor and paracancerous tissues. The results showed that miR-1278 expression level was significantly downregulated in GC tissues (Figure 1a). Furthermore, we detected that miR-1278 expression levels were significantly downregulated in GC cell lines (HGC-27, AGS, and GTL-16) compared to those in control cells GES-1 (Figure 1b). Subsequently, we investigated the role of miR-1278 in miR-1278 mimic-transfected AGS and GTL-16 cells (Figure 1c). CCK-8 analysis confirmed the inhibition of GC cell survival by the miR-1278 mimic (Figure 1d). Moreover, Transwell migration and invasion analysis revealed that the miR-1278 mimic inhibited the migration and invasion of GC cells (Figure 1e–f). Western blotting analysis demonstrated that E-cadherin expression was enhanced by the miR-1278 mimic, whereas the expression of N-cadherin and Snail was impaired by the miR-1278 mimic (Figure 1g). In addition, since the MAPK signaling pathway is essential for both cell proliferation and migration, we analyzed how miR-1278 overexpression affects the MAPK signaling pathway in GC cells. Western blotting analysis showed that the miR-1278 mimic significantly inhibited the relative expression levels of Ras, p-ERK, and p-P38 (Figure 1h). These results suggest that, in GC, miR-1278 levels decreased, and miR-1278 may repress cell survival and migration by inhibiting the MAPK pathway.

### 3.2 miR-1278 overexpression inhibits GC cell growth *in vivo*

**Figure 2:**
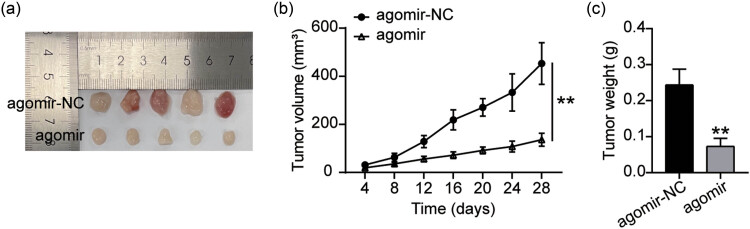
miR-1278 overexpression inhibits GC cell growth *in vivo*. (a) Images of the xenograft tumors in each group 28 days after a subcutaneous injection of AGS cells stably transfected with agomir-NC and agomir. (b) The effect of miR-1278 overexpression on tumor growth in BALB/c nude mice was measured every 4 days (compared with the agomir-NC group, **P < 0.001). (c) The effect of miR-1278 overexpression on tumor weight 28 days after a subcutaneous injection of AGS cells (compared with the agomir-NC group, **P < 0.001).

To clarify the effect of miR-1278 on tumor cell growth *in vivo*, nude mouse xenograft experiments were conducted. The results showed a significant inhibition in tumor xenograft size in the miR-1278 agomir compared to the agomir-NC group (Figure 2a). In addition, tumor growth curves showed that tumor volume increased with the extension of xenograft time but decreased in the agomir compared to the agomir-NC group (Figure 2b). Furthermore, as shown in Figure 2c, the tumor weights in the xenograft model mice were significantly reduced by miR-1278 overexpression. These data confirm the suppression effect of miR-1278 on GC cell growth *in vivo*.

### 3.3 CALD1: a putative target of miR-1278

**Figure 3:**
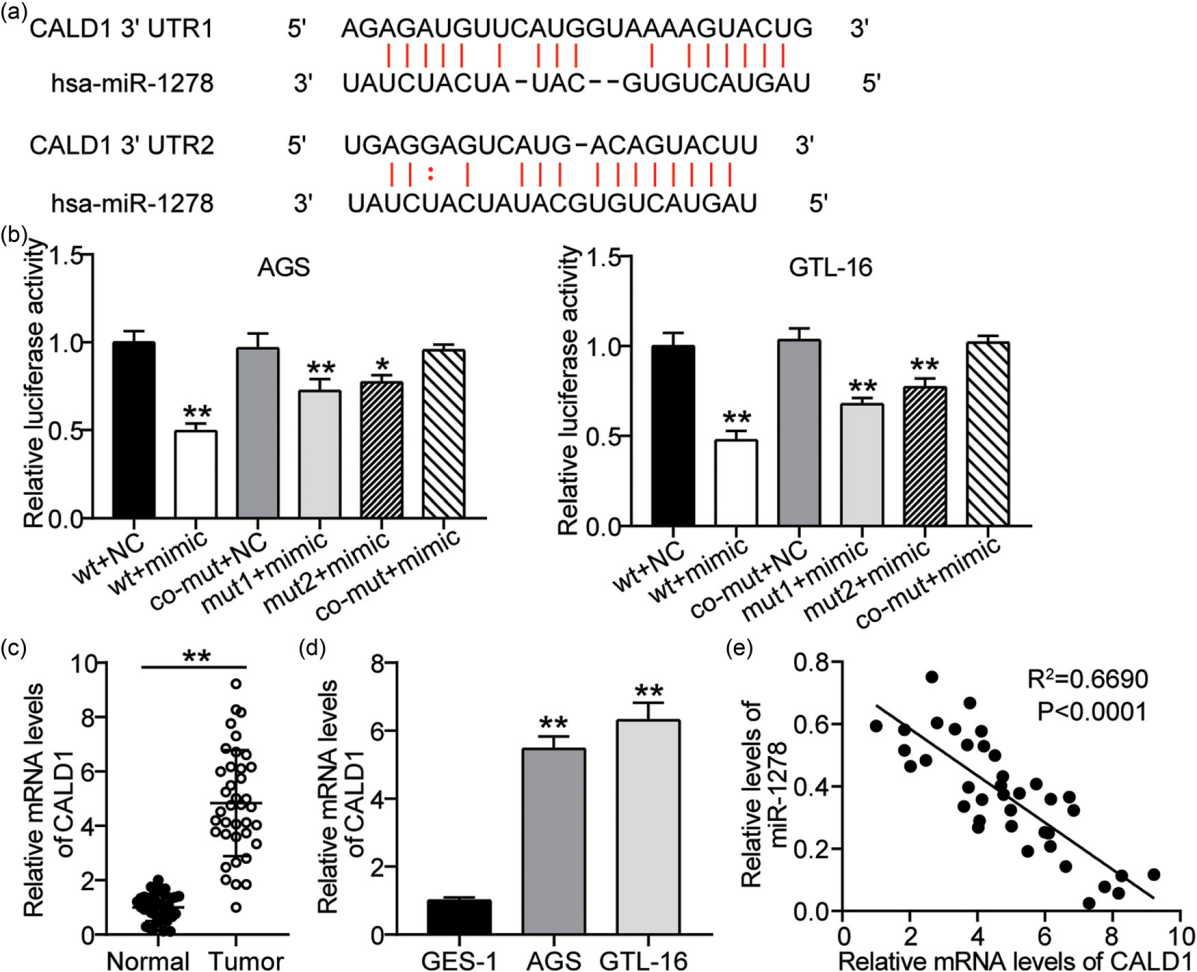
CALD1: a putative target of miR-1278. (a) CALD1 was predicted as a miR-1278 target gene using the starBase website. (b) The targeting relationship between miR-1278 and CALD1 was estimated using a luciferase assay (compared with the wt + NC group, *P < 0.05 and **P < 0.001). (c) The expression levels of CALD1 in GC and paracancerous tissues were estimated using qRT-PCR (**P < 0.001). (d) The expression levels of CALD1 in GC cells (AGS and GTL-16) and GES-1 were estimated using qRT-PCR (compared with the GES-1 group, **P < 0.001). (e) In GC tissues, Pearson’s correlation was performed to assess the relationship between miR-1278 and CALD1 expressions.

The above results suggest that miR-1278 has a tumor-suppressive role on the survival and migration of GC cells. Next, we explored the downstream target genes of miR-1278. We had previously predicted the downstream target genes of miR-1278 using starBase and further identified that miR-1278 had two binding sites for CALD1, using the starBase website (Figure 3a). The dual luciferase reporter assay showed that the luciferase activity in the wt + mimic, mut1 + mimic, and mut2 + mimic groups reduced by approximately 55, 30, and 25%, respectively, compared to that in the wt + NC group, while no significant change was detected in the co-mut + mimic group (Figure 3b). We also found that CALD1 levels increased in tumor tissues (Figure 3c) and GC cell lines (Figure 3d) compared to those in paraneoplastic tissues and the GES-1 cell line, respectively. Furthermore, Pearson’s analysis revealed that miR-1278 and CALD1 levels were inversely correlated in the tumor tissues (Figure 3e). These results show that CALD1 is highly expressed in GC tissues and cells and is negatively correlated with miR-1278.

### 3.4 Restoration of CALD1 reverses the miR-1278 effects on both GC cell growth and the MAPK signaling cascade

**Figure 4:**
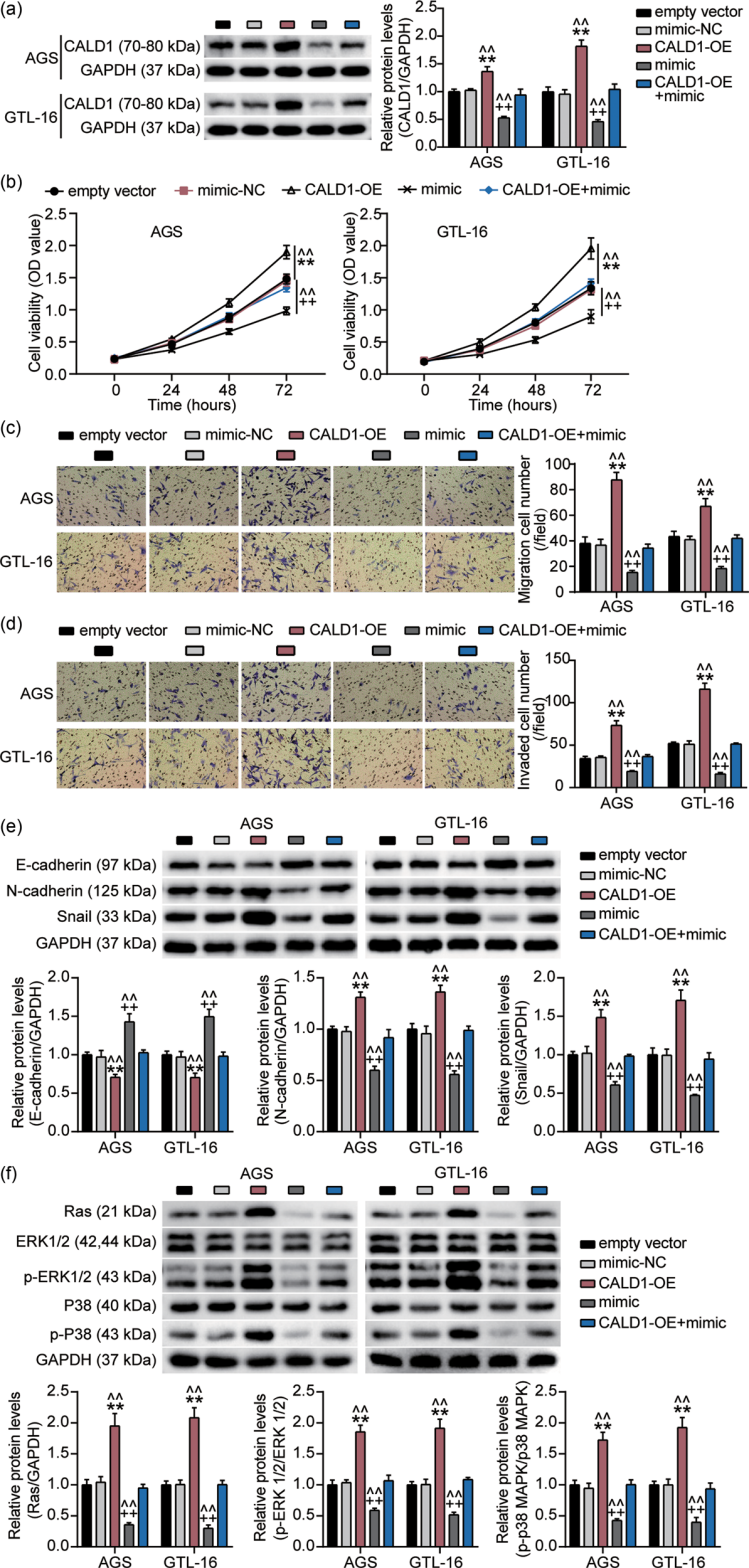
Restoration of CALD1 reverses the effects of miR-1278 on GC cell growth and the MAPK signaling cascade. (a) Western blotting analysis of the CALD1 protein expression in CALD1-overexpressed AGS and GTL-16 cells transfected with an miR-1278 mimic. (b) Cell viability in CALD1-overexpressed AGS and miR-1278 mimic-transfected GTL-16 cells was analyzed using a CCK-8 kit. (c and d) Cell migration and invasion in CALD1-overexpressed AGS and miR-1278 mimic-transfected GTL-16 cells were analyzed using a Transwell assay. (e) The levels of migration-related proteins (E-cadherin, N-cadherin, and Snail) in CALD1-overexpressed AGS and miR-1278 mimic-transfected GTL-16 cells were analyzed using western blotting. (f) The levels of the MAPK signaling pathway-related proteins (Ras, P38, p-P38, ERK1/2, and p-ERK1/2) in CALD1-overexpressed AGS and miR-1278 mimic-transfected GTL-16 cells were analyzed using western blotting (compared with the empty vector group, **P < 0.001; compared with the mimic-NC group, ^++^ *P* < 0.001; compared with the CALD1-OE + mimic group, ^^^^ *P* < 0.001).

**Figure 5:**
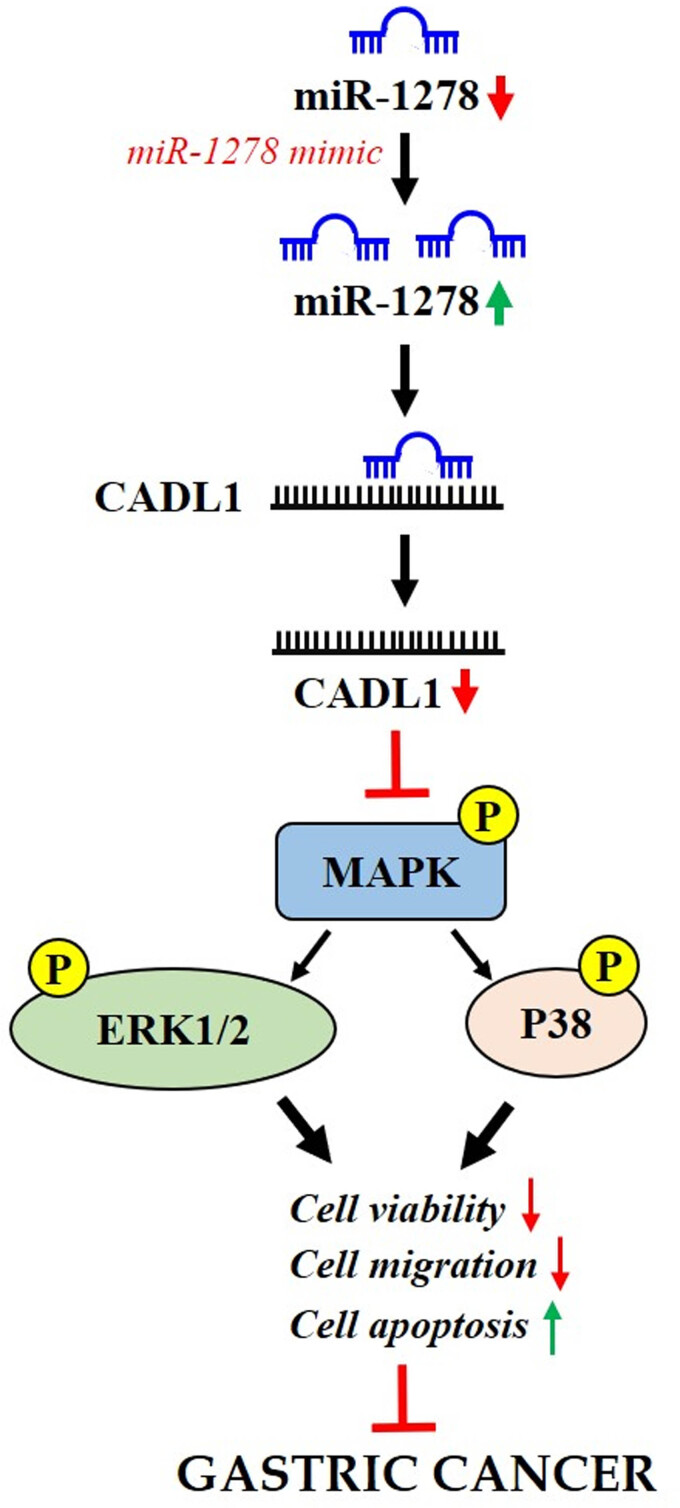
Mechanism of function of the miR-1278/CALD1 axis in GC.

Based on the abovementioned results, we explored the regulatory effect of miR-1278 on CALD1. Western blotting indicated that miR-1278 overexpression significantly inhibited CALD1 protein expression, whereas upregulated CALD1 levels enhanced CALD1 expression and reversed the effect of miR-1278 overexpression (Figure 4a). We then found that CALD1 overexpression significantly promoted cell survival, migration, and invasion and activated the MAPK signaling pathway. The promoting effect of CALD1 overexpression on GC cell survival, migration, and invasion along with the activation of the MAPK signaling pathway was rescued by the miR-1278 mimic (Figure 4b–f). Taken together, our results indicate that miR-1278 suppresses GC progression by targeting CALD1 and inhibiting the MAPK signaling pathway (Figure 5).

## 4 Discussion

GC is among the most widely spread malignancies with a low overall survival rate [18]. The results of our experiments showed that the expression level of miR-1278 was downregulated in GC, whereas miR-1278 overexpression inhibited GC cell survival and migration and suppressed the MAPK signaling pathway. In addition, we found that CALD1 was enriched in GC, and miR-1278 targeted and negatively regulated CALD1 expression. CALD1 overexpression effectively promoted GC cell survival and migration and activated the MAPK signaling pathway. Importantly, the miR-1278 mimic rescued the facilitating effects of CALD1 overexpression on GC cell growth.

It is well established that miRNAs are key factors that influence cancer development. A large number of miRNAs have been found to be differentially expressed and play oncogenic and carcinogenic roles in GC [19]. Several studies have shown that miR-1278 acts as a key regulator in the development of several cancers, including nasopharyngeal and colorectal cancers. In nasopharyngeal carcinoma, miR-1278 exerts tumor-suppressive effects by targeting *ATG28*; miR-1278 expression is downregulated in colorectal carcinoma and tumor cell metastasis is inhibited by the targeting of *CYP24A1*, *BTG2*, and other downstream target genes [11,20]. Interestingly, miR-1278 is enriched in thyroid cancer, whereby it regulates cell proliferation and invasion by targeting *LATS1* [10], suggesting a heterogeneous role of miR-1278 in tumor cells. In our study, miR-1278 expression was found to be downregulated in GC cells, whereas overexpression of miR-1278 inhibited tumor cell survival and migration and suppressed MAPK signaling activation. Additionally, miR-1278 overexpression inhibited tumor growth *in vivo*, which implies that miR-1278 can inhibit GC cell development.

MiRNAs negatively regulate the expression of their target genes by targeting the 3′UTR of the target mRNAs [21]. Here, using bioinformatics prediction coupled with a dual-luciferase reporter assay, we first confirmed that miR-1278 targets and regulates the expression of CALD1, a cytoskeleton-associated protein that is crucial for various cellular functions including proliferation, apoptosis, and adhesion [22,23]. Previous studies have found that CALD1 expression is correlated with the prognosis of gastrointestinal and lung cancer and glioma [24,25]. However, CALD1 also plays variable regulatory roles in different tumors, such as glioma, where it influences tumor progression by regulating tumor angiogenesis; increased CALD1 expression has been associated with poorer prognosis in GC but with better prognosis in lung cancer [16,26,27]. This shows that CALD1 plays a complex regulatory role in tumors. Previous studies have demonstrated high CALD1 expression in GC accompanied by increased immune cell infiltration, suggesting that CALD1 may influence GC progression by regulating the immune microenvironment in tumor cells [16]. Our study indicated that CALD1 enrichment can promote GC cell survival and migration, in addition to activation of MAPK signaling, and this regulatory effect could be rescued by the overexpression of miR-1278. In this sense, we identified a miRNA capable of targeting and negatively regulating CALD1 in GC, which further expands our understanding of GC pathogenesis.

Although we explored the complex pathogenesis of GC in some depth, some limitations remain. First, the correlation between miR-1278 and survival prognosis in patients with GC and cancer histopathology needs to be further studied. Second, the upstream regulatory mechanism of the miR-1278/CALD1 axis warrants further study.

In conclusion, we identified decreased miR-1278 expression and increased CALD1 expression in GC tissues and cells. MiR-1278 directly targets CALD1 and negatively regulates its expression. Overexpression of miR-1278 inhibited cell survival and migration and suppressed the MAPK signaling pathway activation. Moreover, miR-1278 overexpression rescued the promoting effect of CALD1 overexpression on GC progression. The findings of this study provide new insights into the complex regulatory mechanisms in GC and offer new strategies for treating GC.
